# Visualizing Proteasome Activity and Intracellular Localization Using Fluorescent Proteins and Activity-Based Probes

**DOI:** 10.3389/fmolb.2019.00056

**Published:** 2019-08-20

**Authors:** Sabine Schipper-Krom, Alicia Sanz Sanz, Emma J. van Bodegraven, Dave Speijer, Bogdan I. Florea, Huib Ovaa, Eric A. Reits

**Affiliations:** ^1^Department of Medical Biology, Academic Medical Center, University of Amsterdam, Amsterdam, Netherlands; ^2^Department of Medical Biochemistry, Academic Medical Center, University of Amsterdam, Amsterdam, Netherlands; ^3^Leiden Institute of Chemistry, Leiden University, Leiden, Netherlands; ^4^Department of Cell and Chemical Biology, Leiden University Medical Center, Oncode Institute, Leiden, Netherlands

**Keywords:** proteasome, dynamics, fluorescence, activity probes, living cells

## Abstract

The proteasome is a multi-catalytic molecular machine that plays a key role in the degradation of many cytoplasmic and nuclear proteins. The proteasome is essential and proteasome malfunction is associated with various disease pathologies. Proteasome activity depends on its catalytic subunits which are interchangeable and also on the interaction with the associated regulatory cap complexes. Here, we describe and compare various methods that allow the study of proteasome function in living cells. Methods include the use of fluorescently tagged proteasome subunits and the use of activity-based proteasome probes. These probes can be used in both biochemical assays and in microscopy-based experiments. Together with tagged proteasomes, they can be used to study proteasome localization, dynamics, and activity.

## Introduction

Proteasomes are responsible for the degradation of a wealth of proteins in the cell and as such they are essential for many cellular processes. Besides clearing damaged, misfolded, and aged proteins in order to maintain homeostasis, proteasomes also have an important regulatory function in various cellular processes such as transcription and cell cycle control (Hershko and Ciechanover, 1998; Rock and Goldberg, 1999; Glickman and Ciechanover, 2002; Naujokat and Hoffmann, 2002; Geng et al., 2012; Mocciaro and Rape, 2012). Proper proteasome function is thus crucial for cellular viability (Heinemeyer et al., 1991; Velichutina et al., 2004). Additionally, proteasomes are also key players in antigen processing, generating peptides which can be further processed for antigen presentation by MHC class I. Through this process, which can be accomplished either by cleavage or splicing of a protein substrates, proteasomes contribute directly to immune responses against cancer and infection but also autoimmune reactions (Kloetzel, 2004a; Sijts and Kloetzel, 2011). Given the central role of the proteasome in protein homeostasis it is not surprising that it plays a role in the pathogenesis of many diseases, either as primary cause or in secondary responses (Glickman and Ciechanover, 2002; Ciechanover and Brundin, 2003; Ciechanover, 2006; Dahlmann, 2007). Finally, proteasome inhibitors are established therapeutic agents in cancer therapy and are considered for stroke treatment and as immune regulatory agents (Elliott et al., 2003; Kane et al., 2003; Zhang et al., 2006, 2012; Kisselev et al., 2012). Therefore, it is very important to have proper tools at our disposal that allow us to study proteasome function.

**Figure 1:**
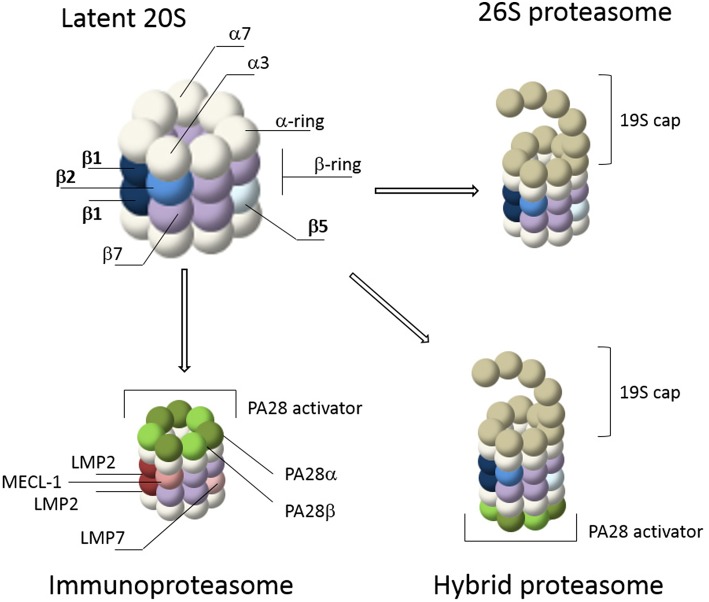
Proteasome composition. The 20S core of the proteasome consists of 4 stacked rings. The outer rings contain seven α-subunits (white) while the inner rings contain seven β-subunits (purple). The catalytic subunits, β1, β2, and β5, are depicted in shades of blue. Gate opening of the 20S core occurs via capping by proteasome activators such as the 19S cap or PA28. The 19S cap is the most abundant activator and it forms the 26S proteasome together with the 20S core. This is a simplified illustration of the 26S cap, a more detailed representation is reviewed elsewhere (Lander et al., 2012; Collins and Goldberg, 2017). IFN-γ stimulation induces *de novo* formation of immunoproteasomes, including 26S immunoproteasomes, containing the immune subunits β1i (LMP2), β5i (LMP7), and β2i (MECL-1) (shades of red), as well as proteasome activation by PA28αβ (shades of green). Together these caps form hybrid proteasomes. In addition, proteasomes can also form complexes with the nuclear activation caps PA200 and PA28γ (not shown).

The core of the proteasome consists of a symmetrical cylinder-shaped structure composed of four stacked rings, each containing 7 different subunits (Figure 1; Puhler et al., 1992; Lowe et al., 1995) and is called the 20S proteasome. The two outer rings are each composed of seven α-subunits (α1-α7 or PSMA1-7). During proteasome assembly, the α-rings serve as backbone for the incorporation of β-subunits, followed by dimerization of two half proteasomes (Coux et al., 1996). In mature proteasomes, the α-rings regulate substrate entrance since the α-subunits have hydrophobic loops that close the 20S barrel to prevent random entry of substrates. In general, protein entry can only be established after gate opening by proteasome activators (PAs) such as the 19S cap, after which substrates can enter the interior of the 20S core for degradation (Voges et al., 1999; Rechsteiner and Hill, 2005; Tanaka, 2009; Lander et al., 2012; Gu and Enenkel, 2014; Collins and Goldberg, 2017). The inner two rings of the 20S barrel consist of the subunits β1-β7 (PSMB1-7). Each β-ring contains 3 catalytic subunits; termed β1, β2, and β5. In mature 20S complexes, the pro-peptides of these catalytic β-subunits are auto-catalytically removed. Upon autocatalytic processing, the N-terminal threonine residues become exposed as the catalytically reactive residues, harboring both the nucleophile (the hydroxyl group) and the catalytic base (the N-terminal amine) involved in peptide bond cleavage (Lowe et al., 1995; Seemuller et al., 1995; Kisselev et al., 2000). Each catalytic subunit has selectivity toward specific residues. β1 has caspase- or peptidyl-glutamyl peptidase-like activity, preferring cleavage at the C-terminus of acidic residues. β2 has trypsin-like activity and cleaves after basic residues, while β5 has chymotrypsin-like activity and prefers cleavage after hydrophobic residues (Orlowski and Michaud, 1989; Heinemeyer et al., 1997).

Proteasome activity can be altered by cytokines such as interferon gamma (IFN-γ). IFN-γ induces the expression of various components of the MHC class I pathway, including the three catalytic immunosubunits β1i (LMP2), β2i (MECL-1) and β5i (LMP7) which replace their constitutive counterparts, β1 β2 and β5 respectively, to form *de novo* immunoproteasomes (Figure 1) (Driscoll et al., 1993; Aki et al., 1994; Groettrup et al., 1995). After stimulation by IFN-γ, immunoproteasomes have a distinct substrate preference and as a result different MHC-class I epitopes are generated (Kloetzel, 2004b; Heink et al., 2005; Seifert and Kruger, 2008; Huber et al., 2012). In addition, the induction of immunoproteasomes is not only a consequence of the immune response, but can also result from oxidative stress (Li et al., 2010; Pickering et al., 2010, 2012; Seifert et al., 2010). IFN-γ also induces expression of the proteasome activator PA28αβ. This regulatory particle controls peptidase activity by opening the 20S barrel, allowing large peptides and unstructured protein domains to enter for degradation (Realini et al., 1997; Rechsteiner and Hill, 2005; Cascio, 2014). Control of proteasomes by PA28αβ was initially thought to specifically increase the production of peptides for MHC class I antigen presentation (Groettrup et al., 1996; Rechsteiner et al., 2000; Cascio et al., 2001). However, more recently it was suggested that PA28αβ acts as a sieve that only selectively releases longer peptides based on their size and sequence (Raule et al., 2014). In addition to the 19S and PA28αβ regulatory particles, 20S proteasomes can also be regulated by the nuclear activators PA28γ and PA200 (Mao et al., 2008; Tanaka, 2009; Savulescu and Glickman, 2011; Huang et al., 2016). A combination of the 20S proteasome with two different regulators, such as the 19S and PA28 regulatory particles, is called a hybrid proteasome (Tanahashi et al., 2000; Bousquet-Dubouch et al., 2011). Finally, proteasome activity can also be regulated by other interacting proteins and by specific post translational modifications (PTMs) (Guo et al., 2017; VerPlank and Goldberg, 2017; Lee et al., 2018; Sbardella et al., 2018).

**Figure 2:**
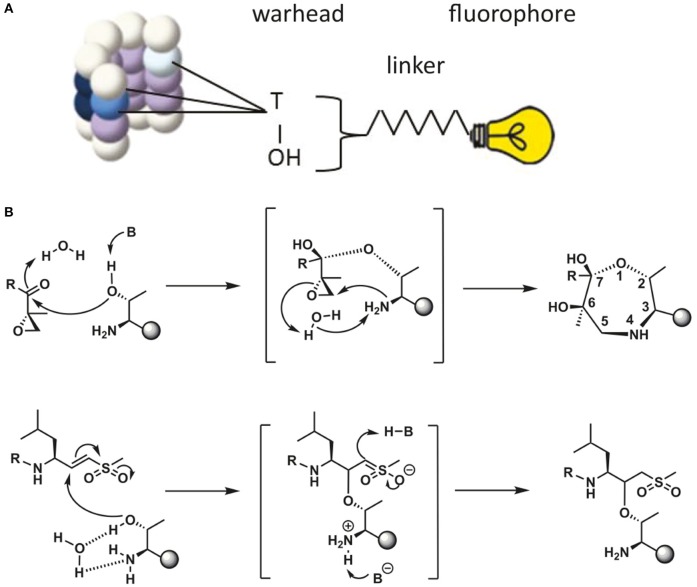
Schematic representation of ABP reaction mechanism. **(A)** Schematic representation of a proteasome activity-based probe (ABP). Labeling of active proteasomes occurs via a nucleophilic attack of the proteasome active threonine residue at the electrophilic trap of the ABP, which in turn captures the catalytic threonine via a covalent bond. A fluorophore can be connected to the probe via a linker for visualization. **(B)** Reaction mechanisms of epoxyketone (upper) (Borissenko and Groll, 2007; Schrader et al., 2016) and vinyl-sulphone (lower) (Borissenko and Groll, 2007). Electrophilic traps react with the N-terminal Threonine residue of the proteolytically active β-subunits. The sphere represents the remainder of the β-subunit. A seven membered ring has been observed by crystallographic methods for the epoxyketone. The vinyl sulphone creates a single covalent ether bond with the N-terminal threonine nucleophile.

Proteasome activity can be detected by taking advantage of activity-based probes (ABPs). Over the last two decades these ABPs have been fine-tuned to improve their potency, selectivity and ease of activity detection (Kessler et al., 2001; Berkers et al., 2005; Verdoes et al., 2006). The general principle of ABP function is shown in Figure 2, with the warhead being a chemical reactive group that covalently binds to the catalytic N-terminal threonine oxygen nucleophile of the proteolytic 20S subunits (Verdoes et al., 2009). ABPs react with proteasomes in a way that corresponds to their catalytic activity and because of their fluorescent properties, they can be imaged specifically and sensitively in cell lysates after gel-electrophoresis followed by fluorescent scanning or in living cells by fluorescence microscopy. Important drawbacks of these probes is that they act as inhibitors as they irreversibly bind the catalytic sites and that they are unable to detect altered substrate recognition and degradation by the proteasome (Table 1).

**Table 1:** Overview of fluorescent activity tools.

**Application/properties**	**Activity binding probe**	**Fluorogenic (quenched) peptides**	**UPS reporters**
20S activity	x	x	
Ubiquitin dependent degradation			x
Catalytic subunit identification	x		
Complex identification	x	x	
Localization	x		
Kinetics		x	
Labeling	x		
Cell permeable	x		
In gel activity analysis	x	x	
FACS analysis	x		

*Based on the question, a proper tool should be chosen to study proteasome function. This overview can be used to determine which tool can be used best to study proteasomes*.

In this review we give an overview of various methods that can be used to visualize proteasomes. We discuss methods that allow one to determine the intracellular distribution of active proteasomes, and to determine changes in proteasome activity either in intact cells or in cell lysates. We also describe methods that allow the determination of the efficiency by which fluorescently tagged subunits are incorporated into proteasome complexes. Such tagged proteasomes are key in studies of proteasome distribution and localization of proteolytic activity.

## Materials and Methods

### DNA Constructs

α3-GFPn1 and β7-mRFP were kindly provided by Prof. N.P. Dantuma (Karolinski Institute, Stockholm, Sweden). Annealing the forward primer 5′-CCGGTATTTCTTAATTGTTGTCCTGGTTGTTGTATGGAACCTTAAT-3′ and the reverse primer 5′-CTAGATAAAGGTTCCATACAACAACCAGGACAACAATTAAGAAATA-3′ resulted in a C4-tag with flanking restriction sites for AgeI and XbaI. GFP was removed using the same restriction enzymes and the annealed tag was inserted. β7-GFP was generated by obtaining β7 via PCR from the mRFP backbone using the following primers: forward 5′-GCGGAATTCCCACCATGGAAGCGTTTTTGGGG-3′ and reverse 5′-GGGCCCTTCAAAGCCACTGATGATG-3′. The PCR product was cloned into an eGFPn2 vector using ApaI and EcoRI. β7-C4 was generated by annealing the forward primer 5′-CTTTCTTAATTGTTGTCCTGGTTGTTGTATGGAACCTTAGGC-3′ and the reverse primer 5′-GGCCGCCTAAGGTTCCATACAACAACCAGGACAACAATTAAGAAAGGGCC-3′ which results in a C4-tag with flanking restriction sites for ApaI and NotI. GFP was removed using the same restriction enzymes and the annealed tag was inserted. α7-GFP was kindly provided by Dr. O. Coux (CRBM Institute, Montpellier, France). Generation of Ub-Q99 and Ub-Q99-C4 was described earlier (Gillis et al., 2013).

### Cell Culture and Transfection

β1i-GFP and β5i-GFP were transfected in HeLa cells with polyethylenimine and kept on G418 selection, 750 mg/ml (GIBCO/Invitrogen, Breda, The Netherlands) in DMEM supplemented with 10% fetal calf serum (FCS) and penicillin/streptomycin/L-glutamine (GIBCO/Invitrogen, Breda, The Netherlands). a3-GFP and a7-GFP in U2OS (kindly provided by Dr. O. Coux, CRBM Institute, Montpellier, France) were kept in DMEM supplemented with 10% FCS and penicillin/streptomycin/L-glutamine (GIBCO/Invitrogen, Breda, The Netherlands). HeLa, U2OS, and HEK293 cells were also cultured in DMEM supplemented with 10% fetal calf serum and penicillin/streptomycin/L-glutamine (GIBCO/Invitrogen, Breda, The Netherlands). All cell lines were kept at 37°C in a 5% CO_2_ atmosphere. HeLa, U2OS, and HEK293 cells were transfected with jetPEI as described by the manufacturer (Polyplus transfection). For confocal microscopy imaging, cells were grown on 2 cm coverslips (Menzel Glaser, Braunschweig, Germany) in 6-well plates.

### Native Gel Analysis

HEK293 cells were harvested in TSDG buffer (10 mM Tris pH 7.5, 25 mM KCl, 10 mM NaCl, 1.1 mM MgCl_2_, 0.1 mM EDTA, and 8% glycerol) and lysed by 3 freeze/thaw cycles in liquid nitrogen. After centrifugation (15 min, 20.817x *g*) the concentration of the supernatant was determined by Bradford protein assay (Serva, Heidelberg, Germany). 4x Native sample buffer (20 mM Tris pH 8.0, 50% glycerol, 0.1% bromophenol blue) was added to 25 μg lysate. The samples were loaded on a 4–12% Creterion XT Precast Bis-Tris gel (Biorad, Hercules, CA, USA) and ran for 3–4 h at 180 V. Fluorescence imaging was performed on a Typhoon Trio (GE Healthcare) using 520 BP 40 filter for GFP detection. For Western blotting, native gels were transferred to a PVDF membrane (Millipore, Bedford, MA, USA) in transfer buffer (25 mM Tris pH 7.5, 192 mM Glycine, 20% MeOH) using the Creterion blotter (Biorad, Hercules, CA, USA). α2 proteins were detected by the MCP236 antibody (1:1,000, kindly provided by Prof. R. Hartmann-Petersen, Biologisk Institut, University of Copenhagen, Copenhagen), PA28α antibodies were directed against the epitope RVQPEAQAKVDVFRED [1:3,000, kindly provided by Prof. M. Groettrup, University of Konstanz, Germany (Macagno et al., 2001)] and antibody detection was done by the Odyssey detection system (LICOR Bioscienses, Lincoln, NE, USA).

### Biarsenical Labeling, Confocal Imaging, and Photobleaching

At 48 h after transfection, HeLa cells were stained as described by Martin et al. (2005) to stain the pre-existing pool of C4-tagged proteins. Briefly, 1 mM ReAsH was pre-incubated in 10 mM 1,2- ethanedithiol (EDT, Sigma*-*Aldrich, Steinheim, Germany*)* in dimethyl sulfoxide (DMSO) for 10 min. Subsequently, cells were washed using PBS (GIBCO/Invitrogen, Breda, The Netherlands) and incubated for 45 min at 37°C with 1 μM ReAsH in Optimem (GIBCO/Invitrogen, Breda, The Netherlands), followed by 4 washes at RT in wash medium (complete DMEM medium with 1 mM EDT). Subsequently, cells were incubated at 37°C for 8 h in the presence or absence of 50 μM cycloheximide (Sigma, St. Louis, MO, USA) or for 20 h in DMEM supplemented with 20% fetal calf serum. After the chase, newly synthesized proteins were labeled with FlAsH by the same procedure. Following the washing steps, cells were harvested using trypsin, washed in PBS, resuspended in lysis buffer (50 mM Tris pH 7.4, 250 mM Sucrose, 50 mM MgCl, 5 mM DTT, 2 mM ATP) and lysed by 3 freeze/thaw cycles in liquid nitrogen. After centrifugation (15 min, 20.817x g) native sample buffer (20 mM Tris pH 8.0, 50% glycerol, 0.1% bromophenol blue) was added and the total supernatant was directly added to a 3–12% NativePAGE Novex Bis-Tris Gel (Invitrogen, Life Technologies Europe BV, Bleiswijk, The Netherlands). Electrophoresis was performed at 150 V for 3 h. Fluorescent detection was done on a Typhoon Trio (GE Healthcare) using the 610 BP 30 filter to detect ReAsH and the 520 BP 40 filter for FlAsH detection. Proteins were transferred and blotted as described above. For confocal imaging cells were fixed with 4% paraformaldehyde (EMS, Hatfield, PA, USA) in 1x PBS (GIBCO/Invitrogen, Breda, The Netherlands) after washing steps and embedded in Vectashield containing DAPI (Vector Laboratories, Burlingame, CA, USA). Samples were examined using a Leica TCS SP8 confocal microscope equipped with UV (405 nm), Argon (488 nm), and a white light laser (e.g., for ReAsH excitation) and 40x or 63x objective (Leica Microsystems, Mannheim, Germany). For the described fluorescence loss in photobleaching (FLIP) experiments, an averaged image was obtained prior to Photobleaching, followed by 25 scans with 100% Argon laserpower and FRAP booster enabled. The entire cytoplasm was selected as region of interest (and the entire nucleus excluded). During bleaching and immediately after bleaching, the fluorescence of the nucleus was monitored by time-lapse imaging. The remaining fluorescence in perspective to the cytoplasm was quantified and defined as the ‘immobile fraction’ (proteins too large to diffuse passively into the cytoplasm).

### Activity Labeling in Living Cells

U2OS cells were incubated for 1 h at 37°C in serum free medium supplemented with 0.5 μM ABP1 (green BodipyFL-Ahx_3_L_3_VS) (Berkers et al., 2007), ABP2 (green Bodipy-epoxomicin, LW66) (generated by Prof. HS. Overkleeft, Leiden Institute of Chemistry and Netherlands Proteomics Centre, The Netherlands), ABP3 (red BodipyTMR-Ahx_3_L_3_VS,) (Verdoes et al., 2006), and ABP4 (yellow Bodipy-Cy3-epoxomicin, MVB003) (Florea et al., 2010). Cells were subsequently washed 3 times in PBS (GIBCO/Invitrogen, Breda, The Netherlands) and imaged using a Leica TCS SP8 X confocal microscope equipped with white light laser and stage incubator (Leica Microsystems, Mannheim, Germany). To determine nonspecific binding, cells were pre-incubated with 1 μM epoxomycin (Sigma, St. Louis, MO, USA) for 1 h. 100 U/ml IFN-γ (Roche diagnostics, Mannheim, Germany) was added to medium to induce immunoproteasomes. Quantifications were done by means of Leica LAS AF light software (Leica Microsystems, Mannheim, Germany).

### Activity Labeling in Lysates

HEK293 cells were harvested in TSDG buffer (10 mM Tris pH 7.5, 25 mM KCl, 10 mM NaCl, 1.1 mM MgCl_2_, 0.1 mM EDTA, and 8% glycerol) and lysed by 3 freeze/thaw cycles in liquid nitrogen. After centrifugation (15 min, 20.817x g) the concentration of the supernatant was determined by a Bradford protein assay (Serva, Heidelberg, Germany). Proteasomes were labeled in lysates using 0.5 μM ABP2 (Bodipy-epoxomicin) (Florea et al., 2010) for 1 h at 37°C. 4x Native sample buffer (20 mM Tris pH 8.0, 50% glycerol, 0.1% bromophenol blue) was added to 25 μg lysate. The samples were loaded on a 4–12% Creterion XT Precast Bis-Tris gel (Biorad, Hercules, CA, USA) and ran for 3–4 h at 180 V. Alternatively, 3–12% NativePAGE Novex Bis-Tris Gels (Invitrogen, Life Technologies Europe BV, Bleiswijk, The Netherlands) were used to identify 26S capped proteasomes. For the detection of individual subunits on SDS-PAGE, 10 μg cell lysate was incubated with 0.5 μM ABP. After 1 h incubation at 37°C, 6x sample buffer (350 mM Tris/HCl pH 6.8, 10% SDS, 30% glycerol, 6% β-mercaptoethanol) was added, samples were boiled and loaded on a 12% SDS-PAGE. Fluorescence imaging was performed on a Typhoon Trio (GE Healthcare) using the 580 BP 30 filter to detect the ABP1 and ABP2, and the 520 BP 40 filter was used for detection of ABP3 and APB4.

### Activity Labeling in 2D

A confluent 10 cm plate of HeLa cells was harvested in 500 μl proteasome-activity buffer (50 mM TRIS pH 7.5, 50 mM Sucrose, 50 mM MgCl, 1 mM DTT, 1 mM ATP). Lysis by 3 cycles of freezing/thawing in liquid nitrogen. Protein concentrations were determined using Bradford (Serva, Heidelberg, Germany), 500 μg final protein concentration was taken and incubated/ with 0.5 μM ABP4 for 1 h at 37°C. TCA precipitation was performed to reduce the sample volume and the protein pellet was dissolved in 125 μl Urea buffer (7.7 M Urea, 2.2 M Thiourea, 4% CHAPS, 30 mM TRIS pH 9.8) with 0.5% hydroxyethyl-disulfide (Destreak reagent, GE healthcare) and 2% IPG buffer (pH 3–10 NL, GE Healthcare) freshly added. The samples were loaded on a Immobiline drystrip (pH 3–10 NL, GE Healthcare) and incubated o/n at room temperature. IEF was performed on a Protean IEF Cell (Biorad, Hercules, CA, USA) using the following program; 0.1 min 50 V, 30 min 200 V; 30 min 200 V, 30 min 400 V, 30 min 400 V, 30 min 600 V, 60 min 3,500 V, 240 min 3,500 V, 10 min 200 V. After focusing, the strips were incubated for 0.5 h in equilibration buffer (50 mM TRIS 8.8, 6 M Urea, 30% (v/v) Glycerol, 20% (w/v) SDS, BPB) with 10 mg/ml fresh DTT. Subsequently, the strips were directly transferred in equilibrium buffer with 25 mg/ml IAA and incubated for 0.5 h. The strips were recovered, loaded on top of a 12% SDS-PAGE gel and fixed in agarose sealing solution (15% v/v glycerol, 1% agarose, 1x Leammli electrophoresis buffer, BPB). Electrophoresis was performed at 30 mA per gel. Fluorescent detection was done on a Typhoon Trio (GE Healthcare) 580 BP 30 filter to detect ABP4.

### Activity Measurements in Gel

HEK293 cells were harvested in TSDG buffer (10 mM Tris pH 7.5, 25 mM KCl, 10 mM NaCl, 1.1 mM MgCl_2_, 0.1 mM EDTA, 8% glycerol, and 1 mM ATP was added freshly), lysed by three freeze/thaw cycles in liquid nitrogen and protein levels were determined by a Bradford assay (Serva, Heidelberg, Germany). 40 μg of cell lysates were incubated with 0.5 μM ABP4, 0.5 μM Epoxomicin (Sigma, St. Louis, MO, USA) or similar amounts of DMSO for 1 h at 37°C. Samples were loaded on a 3–12% NativePAGE Novex Bis-Tris Gels (Invitrogen, Life Technologies Europe BV, Bleiswijk, Netherlands) and run at 150 V for 3 h. For in gel proteasome labeling, the gel was first scanned for fluorescence on a Typhoon Trio imager (Ge Healthcare) using the 520 BP 40 filter. Following this, the wet gel slab was incubated for 20 min in 20 ml Overlay buffer (20 mM Tris pH 7.5, 5 mM MgCl_2_, 1 mM ATP) and 0.25 μM ABP4. After three washes in Destain buffer (5% acetic acid, 20% MeOH) for 10 min, the gel was scanned again for fluorescence to detect additional labeling. To detect substrate cleavage in gel, the wet gel slab was incubated in overlay buffer with 400 nM of the quenched peptides directly after electrophoresis. Fluorescent intensities were measured on a Typhoon Trio imager (GE Healthcare) using the 580 BP 30 filter. The wet gel slabs were transferred to PVDF membrane (Millipore, Bedford, MA, USA) and Western blotting was performed as described above.

## Results

### Visualizing Proteasome Activity

Altered UPS function is related to various diseases. Increased proteasomal degradation was measured in muscle wasting diseases and down-regulation of proteasome function in a wide range of neurodegenerative diseases (Hishiya et al., 2006; Cohen et al., 2015; Bilodeau et al., 2016; Lee et al., 2018; Reddy et al., 2018). Proteasome activity also decreases during aging, which may contribute to various late onset disorders (Breusing and Grune, 2008; Morimoto and Cuervo, 2009). In addition, altered proteasome composition can also induce changes in activity, like the incorporation of immuno subunits or altered capping by PA complexes. Both ubiquitin-independent fluorogenic peptides (Kisselev and Goldberg, 2005) and ubiquitin-dependent fluorescent reporter proteins (Lindsten and Dantuma, 2003) are valuable tools to determine proteasome activities, but cannot be used to visualize the intracellular localization of active proteasomes (Dantuma et al., 2000; Bennett et al., 2005; Hoyt et al., 2005; Kisselev and Goldberg, 2005; Table 1). The more recently developed activity-based probes (ABPs) have often been used to detect alterations in proteasome activity in cell lysates but can also be used to visualize proteasome activity in living cells (Liggett et al., 2010; Li et al., 2012, 2013). ABPs are small molecules consisting of a proteasome inhibitor linked to a small fluorophore. Fluorescence labeling of proteasomes occurs via a nucleophilic attack of the catalytic N-terminal threonine toward the ABP, leading to a covalent, irreversible bond between the warhead of the ABP and the proteasome active site (Borissenko and Groll, 2007; Schrader et al., 2016; Figure 2). Importantly, unlike fluorescently tagged proteasome subunits, as discussed later, the ABPs only label fully assembled, active proteasome complexes. In addition, ABPs were recently also used to label and inhibit transmembrane proteasomes, taking advantage of cell impermeable biotin connected to epoxomicin (Ramachandran and Margolis, 2017).

In this toolbox we use fluorescently labeled ABPs to analyze 20S containing complexes and their activity. We used two types of proteasome probes that have been developed for activity labeling (Figure 2). The first type of probe has a vinyl sulphone warhead and is connected to a Bodipy fluorophore, which we will refer to as **ABP1** (green fluorophore), and **ABP3** (red fluorophore) (Verdoes et al., 2006; Berkers et al., 2007). The second activity probe has an epoxomicin-based warhead which is also connected to a Bodipy fluorophore (Florea et al., 2010; Li et al., 2013). These probes are further referred to as **ABP2** (green fluorophore) and **ABP4** (yellow fluorophore). The vinyl sulphone electrophilic trap connected to a trileucine motif, although originally developed as cysteine protease privileged electrophile, proves to act as an excellent proteasome inhibitor (Bogyo et al., 1997). In order to allow multiplexing, each warhead was appended with fluorophores which are often used in cellular imaging. Due to their differences in chemical structure and lipophilicity, some variation can be seen in the distribution of the different ABPs in living cells, these are due to differences in wash out rates from lipophilic cellular compartments like the ER. In general, there was no a-specific labeling detected when using ABPs. In the past however, one single off-target protein for the vinyl sulphone-based probes was observed. This off-target protein was identified as cathepsin which is only observed in specific tissues (Berkers et al., 2007). This cathepsin is also an off-target of the widely used MG132. For the epoxomicin-based ABPs we have not observed any off-targets so far.

Here we describe methods to determine proteasome activity using ABP labeling in living cells and in cell lysates upon SDS- and Native-PAGE analysis.

#### Proteasome Activity Measurement Upon Labeling in Living Cells

**Figure 3:**
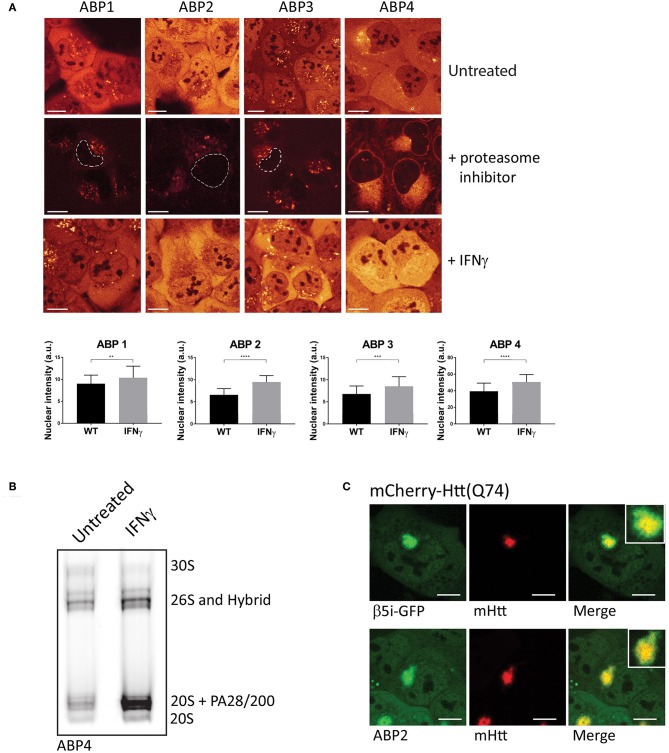
Visualizing proteasome activity in living cells. **(A)** Proteasome activity labeling in living cells by different probes. U2OS cells were incubated with vinyl sulphone (**ABP1**, green and **ABP3**, red) and epoxomicin (**ABP2**, green and **ABP4**, yellow) based probes (upper panel). Pre-incubation with epoxomycin to block proteasome activity was used to determine nonspecific binding (middle panel). Epoxomycin-based probes give more intense labeling pattern, while the Cy3 fluorophore gives more background staining. When U2OS cells were stimulated for 72 h with IFN-γ, subsequent activity labeling showed a significant increase in labeling (lower panel, graphs). **(B)** U2OS cells were stimulated with IFN-γ, labeled with **ABP4** and analyzed by native PAGE, confirming increased ABP labeling as shown by microscopy. **(C)** Recruitment of active proteasomes into aggregates. U2OS cells were transfected with polyglutamine-expanded huntingtin fragments to initiate aggregation, and co-transfected with β5i-GFP to show proteasome distribution around aggregates. Incubation with ABP2 showed a similar distribution pattern as β5i-GFP, indicating the recruitment of catalytically active proteasomes into aggregates. Scale bar = 5 μm.

In order to visualize and compare the distribution of the ABPs in living cells, we incubated U2OS cells with the four different probes and compared activity labeling by confocal microscopy (Figure 3A). While these probes bind within minutes to their targets, the regular incubation time is 20 min to 2 h. All ABPs show a similar typical proteasome distribution pattern with diffuse labeling of the nucleus and cytoplasm, but not the nucleoli (Figure 3A, upper panel). Incubation with proteasome inhibitors prior to probe labeling revealed a less intense non-specific perinuclear staining which was most abundant after incubation with **ABP4** (Figure 3A, middle panel). When cells were incubated with IFN-γ to induce expression of immunoproteasomes, all probes reported significantly increased labeling and hence activity of proteasomes (Figure 3A, lower panel). For the quantification of probe labeling in living cells, we compared fluorescence between untreated and IFN-γ stimulated cells using identical confocal settings. Fluorescence intensity was only measured in the nucleus thereby excluding the non-specific labeling in the cytoplasm as shown in the middle panel. This increased labeling after IFN-γ stimulation is confirmed by native gel analysis (Figure 3B). Alternatively, flow cytometry can be used to quantify ABP labeling in living cells, or *in gel* analysis upon cell lysis as described below.

The **ABPs 1–4** can also be used to examine recruitment of active proteasomes to particular intracellular sites after particular stimuli or conditions. Huntington's disease (HD) is a neurodegenerative disease, hallmarked by the formation of intracellular aggregates induced by polyglutamine (polyQ) expanded huntingtin protein fragments (Ross, 1997). Various studies have suggested that proteasomes which are recruited into polyQ aggregates become impaired by the polyQ fragments (Holmberg et al., 2004; Venkatraman et al., 2004). When cells were transfected with mCherry-tagged Huntingtin (Q74) to initiate aggregation and GFP-tagged β5i, to visualize proteasome distribution patterns, recruitment of proteasomes into aggregates was observed (Figure 3C, upper panel). Importantly, when cells were transfected with mCherry-Huntingtin (Q74) and subsequently labeled with **ABP2**, similar fluorescence labeling of the aggregates was observed as for β5i-GFP, indicating that recruited proteasomes are catalytically active and accessible for substrates (Figure 3C, lower panel). These examples illustrate how activity labeling of proteasomes in living cells can be used to visualize both localization and activity (Schipper-Krom et al., 2014).

#### Proteasome Activity Measurement Upon Labeling in Cell Lysate

Since all active proteasome complexes can be visualized when using ABP labeling, changes in proteasome complex composition can be studied in cell lysate. The *in gel* detection of proteasome complexes can be done either by adding ABPs to living cells prior to lysis, to cell lysates, or by labeling of proteasome complexes in native PAGE gels (**Figure 5A**). The activity of the ABP-labeled β-subunits can then be visualized and quantified after scanning the wet gel slab for fluorescence without further blotting steps. However, differences in labeling efficiencies occur between these 3 labeling methods (Berkers et al., 2007).

**Figure 4:**
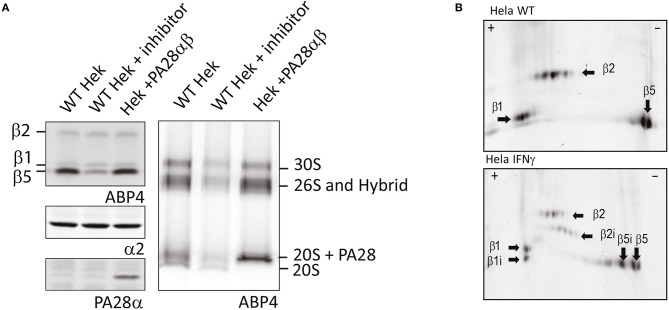
Proteasome activity labeling in cell lysates. **(A)** Activity labeling of individual subunits vs. proteasome complexes. Control cell lysates, lysates pre-incubated with MG132 and lysates of cells overexpressing PA28αβ were incubated with ABP4 and loaded on SDS-PAGE (left) or 3–12% native gels (right). Wet gels slabs were scanned for activity labeling and intensities were determined using AlphaEase software. After transfer to membranes, anti-α2 antibodies were used to identify proteasome complexes and PA28α antibodies were used to show PA28 over-expression. Expression of PA28αβ induced a shift in proteasome activity toward PA28-capped proteasomes. **(B)** Visualizing activity of constitutive and immunosubunits on 2D gels. Cell lysates of control Hela cells and IFN-γ stimulated HeLa cells were incubated with **ABP4**, subjected to pH 3–10 strips to separate proteins in the first dimension. Subsequently, proteins were separated by size in the second dimension on a 12% SDS-PAGE gel. Fluorescence scanning revealed the labeled subunits. Unlike visualization on a one dimention SDS gel, the activity of all six catalytic subunits could be visualized individually.

First, analysis of living cells treated with ABPs on SDS-PAGE shows a very slight background labeling of other proteins, although the major bands with the highest contribution represent proteasome labeling (Verdoes et al., 2006; Florea et al., 2010). While SDS-PAGE analysis is an easy method to visualize the activity of individual subunits, this method has two limitations. The first is the inability to detect proteasome capping by 19S, PA28, or PA200. Changes in PA capping and activity are difficult to detect by SDS-PAGE since the total pool of all proteasome complexes are represented by one single band for each of the catalytic subunits. For example, when lysates of control cells were compared to lysates of cells that overexpressed PA28αβ, no significant differences in proteasome activity were observed upon incubation with **ABP4** (Figure 4A, upper left panel). However, when the same lysates were separated on 3–12% native gradient gels, a shift to PA28-capped and hybrid proteasomes can be observed (Figure 4A, right panel), in parallel with a reduction in 20S proteasomes. Similarly, capping by PA28-γ or PA200 can be established after nuclear extraction.

A second drawback of these probes is that they cannot discriminate between the catalytic constitutive and immunoproteasome subunits. Since β5, β5i, β1, and β1i have similar molecular weights, discrimination by SDS-PAGE is difficult. To distinguish between the two types of subunits one can use probes that specifically label immunosubunits (Li et al., 2013). Alternatively, when interested specifically in 20S catalytic subunits, all catalytic sites can be labeled with ABPs and subsequently separated and visualized by 2D gel analysis. Using this method, subunits are not only separated by size, but also due to differences in isoelectric points. 2D analysis has been intensively used to identify proteasome subunits using antibodies or upon radioactive labeling followed by immunoprecipitation or chromatography (Drews et al., 2007b). However, when proteasome subunits are labeled with ABPs, proteasomes do not have to be purified since only the active subunits will be labeled and thus subsequently visualized after scanning the 2D gel for fluorescence. Furthermore, only one single labeling step will reveal the activity of all 6 catalytic subunits.

When non-treated or IFN-γ stimulated HeLa cells were subjected to 2D analysis, all catalytic subunits could indeed be identified, showing the activities of both the household and induced immunosubunits (Figure 4B). Interestingly, most subunits show various fluorescent dots, which may represent post-translational modifications that affect their isoelectric point. Together, this shows that ABPs can be used to analyze different proteasome complexes using native gels and active subunits using SDS-PAGE analysis or 2D-gel analysis for even greater detail. The use of ABPs requires less steps compared to immunostaining or immunopurification protocols.

#### Proteasome Activity Measurement Upon Labeling in Gel

**Figure 5:**
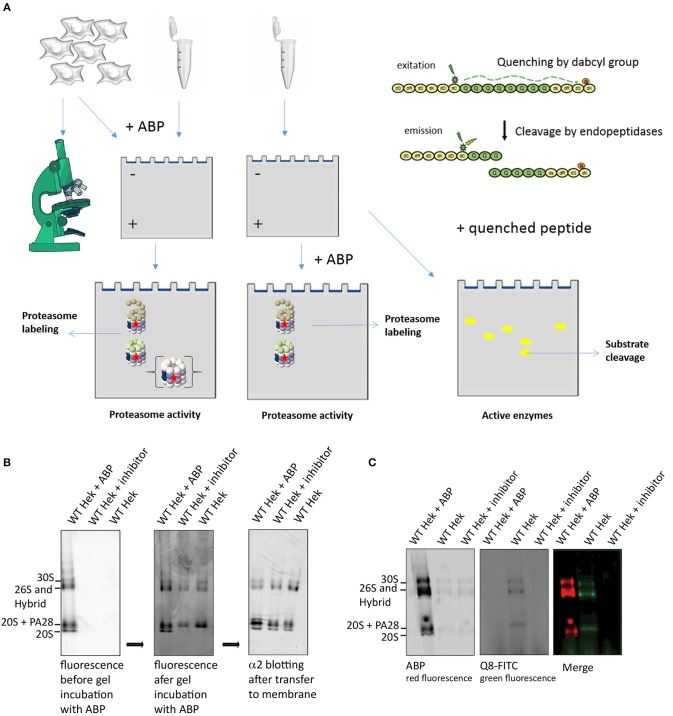
Proteasome activity labeling *in gel*. **(A)** A schematic representation of methods to visualize proteasome activity, either by microscopy or by *in gel* visualization. The left panel represents methods to detect ABP labeling and the right panel explains the use of quenched substrates to determine proteasome specificity. **(B)** Proteasome labeling in gel. HEK293 cell lysate was divided in three fractions, one fraction was pre-incubated with ABP4 for identification of proteasome complexes, one fraction was pre-incubated with proteasome inhibitor to determine specificity and one sample was left untreated. Upon complex separation by a 3–12% native gradient gel, the wet gel slabs were scanned for fluorescence (left panel). Subsequently, the gel was incubated with buffer containing **ABP4** and again scanned for fluorescence (middle panel). After protein transfer to a PVDF membrane, α2-antibodies confirmed the presence of all proteasome complexes in each lysate. Differences in proteasome labeling were observed between the lysates, since in gel labeling only revealed proteasomes capped with a proteasome activator. **(C)** Quenched peptide substrates to determine proteasome specificity in gel. HEK293 cell lysates were divided in three fractions, one fraction was pre-incubated with ABP4 for identification of proteasome complexes, one sample was left untreated and one fraction was pre-incubated with proteasome inhibitor to determine specificity of the fluorescent degradation signal. After complex separation in the gel, the gel was incubated in buffer containing quenched polyglutamine peptides (Q8-FITC) that become fluorescent after cleavage. Merging the two images shows a proteasome cleavage pattern of the Q8-peptide.

Proteasome activity can also be visualized and quantified using *in gel* activity, where active proteasome complexes are separated using native PAGE gels. Subsequent incubation of the gel in a buffer that contains ABPs or fluorogenic substrates results in a local fluorescence signals that can be quantified as a measure of activity (Figure 5A). To demonstrate ABP labeling *in gel*, we pre-incubated cell lysates with either **ABP4**, a proteasome inhibitor or DMSO. Proteasome complexes were separated on 3–12% native gradient gels and subsequently incubated in an ABP containing buffer (Figure 5B). Addition of the ABP prior to or after electrophoresis results in different complex labeling. Addition of ABP to lysates before electrophoresis labels all proteasome complexes including the latent 20S core, whereas addition of ABP after electrophoresis only reveals activated complexes but not the latent 20S complexes. This indicates that ABPs only enter activated proteasome complexes but that a fraction of proteasome complexes dissociate during sample preparation, resulting in labeled 20S complexes (Shibatani et al., 2006). Immunoblotting the α2-subunit to identify the various proteasome complexes, confirmed equal levels of latent 20S in all samples (right panel). This indicates that PAs and 20S can dissociate during sample preparation, leading to the impression that latent 20S proteasomes are labeled by ABPs. However, the presence of potassium chloride in lysates prevents spontaneous activation and diminishes the contribution of the 20S proteasomes (Kohler et al., 2001). In addition, the inability of ABPs to enter 20S propteasomes was previously also confirmed by forced gate closing and opening (Leestemaker et al., 2017).

In addition to ABPs, quenched fluorogenic peptides can also be studied by using this method. The small peptide-based substrates of 3–4 amino acids in length that are attached to a fluorescent group such as 7-amino-4-methylcoumarin (AMC) are often used to detect alterations in the chymotrypsin-like, trypsin-like and peptidylglutamyl-peptide hydrolyzing activities (Kisselev and Goldberg, 2005). These peptides only become fluorescent upon degradation due to separation of quencher and fluorophore that are coupled to different residues (Figure 5A; Reits et al., 2003, 2004; Stargardt and Reits, 2013). However, to examine whether proteasomes can cleave within specific sequences, we developed quenched peptides containing a specific sequence of interest. In this example we used a peptide containing 8 glutamine (Q8) residues as repeated polyQ sequences are related to HD. These amino acids are flanked by non-degradable D-amino acids at both peptide termini to prevent exopeptidase activities and improve solubility. The quencher and fluorophore moieties are coupled to these flanking D-amino acids. When cell lysates were separated on a native gel and incubated with the quenched Q8-peptides, a fluorescence pattern appeared similar to ABP labeled proteasomes (Figure 5C). These bands were not present when lysates were pretreated with proteasome inhibitor, confirming specific degradation of the Q8-peptide by proteasomes. These results illustrate how probes in combination with cleverly designed quenched peptides can be used to detect proteasome activity as well as specificity.

### Studying Proteasome Localization Using Fluorescent Tags

Non-invasive tags, such as green fluorescent protein (GFP), enable visualization of proteasome subunits for studying proteasome distribution and dynamics in living cells, but it is important to ensure that tagged subunits are efficiently incorporated in the proteasome. Large fractions of non-incorporated fluorescent subunits will interfere when studying distribution and kinetics of fluorescently-tagged proteasomes, as their dynamics and localization is different from those which are incorporated, including free diffusion between the nucleus and cytoplasm. GFP-tagged β1i was the first fluorescently-labeled subunit shown to be incorporated in proteasomes (Reits et al., 1997; Groothuis and Reits, 2005). Thereafter, several other fluorescent subunits were used to visualize and study proteasomes in cells (Salomons et al., 2010).

#### Incorporation Efficiency of Fluorescent Proteasome Subunits

The efficiency of subunit incorporation in proteasome complexes can be determined by several means, including immunoprecipitation of proteasome complexes and sucrose gradients. Subsequent immunoblotting for GFP can be used to separate non-incorporated GFP-tagged subunits from those assembled in proteasome complexes (Reits et al., 1997; Enenkel et al., 1998). However, a technique that is easier to perform is proteasome complex separation by native PAGE. This is a simple and straightforward method to examine the level of incorporation of a GFP-tagged subunit into a complex. Since proteins are not denatured upon electrophoresis, all tagged proteins can be visualized directly as GFP remains fluorescent, and the ratio of incorporated vs. non-incorporated fluorescent fusion subunits can then be determined.

**Figure 6:**
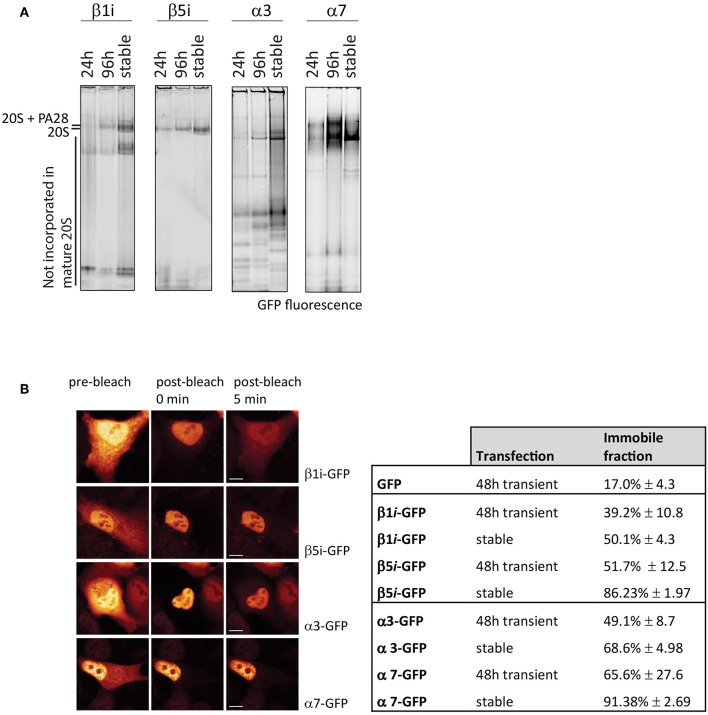
Incorporation of GFP-tagged proteasome subunits. **(A)** Incorporation of transient and stably expressed proteasome subunits. The α-subunits α3-GFP and α7-GFP were expressed in U2OS cells and β1i and β5i were expressed in HeLa cells, either stable or transient for 24 h or 96 h. GFP-tagged subunits were identified by scanning for fluorescence. Stable expression of subunits α3, α7, and β1i did not further improve incorporation when compared to 96 h transient expression. **(B)** Fluorescence loss in photobleaching (FLIP) to distinguish non-incorporated from incorporated GFP-tagged subunits. By photobleaching the entire cytoplasm, the small GFP-tagged subunits that freely diffuse between nucleus and cytoplasm also become photobleached, while GFP-tagged proteasomes in the nucleus remain fluorescent. Cells, either transiently transfected for 48 h or stably expressing the proteasome subunits, were analyzed for free diffusion of non-incorporated subunits by photobleaching the entire cytoplasm and quantifying the decrease in fluorescence in the nucleus. The immobile fraction is the remaining percentage of fluorescence in the nucleus, representing large GFP-tagged proteasome complexes. Nuclear fluorescence of β1i-GFP and to a lesser extent α3-GFP decreases in time, indicating a substantial non-incorporated pool of GFP-tagged subunits (mean ± SD, *N* = 5). Scale bar = 5 μm.

As the position and function of specific subunits differ within the 20S complex, this also affects incorporation efficiencies. To determine differences in incorporation efficiencies between various GFP-tagged α- and β-subunits of the 20S proteasome, we expressed C-terminal tagged α3-GFP (PSMA4), α7-GFP (PSMA3, β1i-GFP (LMP2 or PSMB9), and β5i-GFP (LMP7 or PSMB8) (Figure 6). Only the exposed C-terminus of these subunits is suitable for tagging since the N-terminus of the α-subunits is involved in gating of the 20S barrel, whereas the N-terminal amine residues of the catalytically active β-subunits are essential for proteasome activity (Coux et al., 1996). After 24 and 96 h cells were harvested together with cells that stably express the proteasome subunits. Cell lysates were subjected to 4–12% gradient native gels in order to distinguish proteasome complexes from premature inactive complexes and non-incorporated subunits. After electrophoresis, the wet gel slabs were scanned for GFP fluorescence (Figure 6A). Not all subunits are incorporated with similar efficiencies, as the amount of non-incorporated, faster migrating proteins varied between subunits. While β1i, α3, and also α7 remain partially present in pre-complexes, in both the transient and stable expressing cells, stable expression of β5i results in complete incorporation. This result shows that GFP fluorescence does not necessarily represent intracellular distribution of active proteasomes, since fluorescent pre-complexes do not represent mature and active proteasomes.

Various studies have used β1i-GFP to determine proteasome localization since it has been shown that this subunit is incorporated into active proteasomes, but even stable expression of subunits does not guarantee efficient incorporation into active proteasome complexes. Here the tagged β5i is most representative for studying active proteasome complexes which may be explained by the strong interaction with the proteasome maturation protein (POMP), a chaperone in proteasome assembly (Kruger et al., 2001; Heink et al., 2005). Since we observed GFP-tagged proteasomes being part of larger proteasome complexes than the 20S core alone (data not shown), it can be assumed that the tag does not prevent complex formation with proteasome activators such as the 19S complex. However, it is unknown whether it can only form single capped or also double capped proteasomes, as a single GFP tag may oppose complex formation on that side of the proteasome but not toward the other end of the 20S complex. Importantly, the fluorescent tag does not seem to influence activity as β1i-GFP incorporation did not affect proteasome activity toward fluorogenic peptide substrates (Reits et al., 1997).

#### Photobleaching Techniques to Visualize Proteasome Complex Formation

Fluorescent photobleaching techniques can be used to study the dynamics of fluorescent fusion proteins in living cells. By depleting fluorescence in selected intracellular regions and imaging fluorescence recovery afterwards, mobility of fluorescent proteins can be determined. The most frequently used technique is Fluorescence Recovery After Photobleaching (FRAP), where a small region is briefly illuminated with high laser power and the recovery of fluorescence in this region is monitored in time (Lippincott-Schwartz et al., 2001; Reits and Neefjes, 2001). While mobile, irreversibly photobleached proteins will move out of the monitored region, fluorescent proteins from surrounding regions will move into the bleached area. The rate and level of recovery are directly linked to the velocity and mobility of the proteins, respectively. Alternatively, a specific compartment of the cell can be repeatedly photobleached for a prolonged period, and in time the Fluorescence Loss In Photobleaching (FLIP) can be monitored in another region of the cell. This provides information on trans-compartment movement of the fluorescent proteins between nucleus and cytoplasm, or between different organelles.

When studying proteasome complex formation, the nuclear pore complex can be used as a molecular sieve to distinguish large proteasome complexes from smaller pre-complexes. The nuclear pore allows free diffusion of proteins up to 60–110 kDa, thereby preventing passive diffusion of proteasome complexes (Silver, 1991; Wang and Brattain, 2007). Thus, when applying FLIP, a decrease in fluorescence in the non-bleached compartment represents diffusion of small pre-complexes and non-incorporated subunits between nucleus and cytoplasm. Little or no decrease in fluorescence in non-bleached compartments indicates that the fluorescent subunits are mostly present in large proteasome complexes. Importantly, photobleaching of nuclear regions affects cytoplasmic fluorescence levels too, as the vertical laser beam will also bleach the cytoplasm above and below the nucleus. Therefore, photobleaching the cytoplasm is preferred.

To determine incorporation of GFP-tagged subunits, fluorescence was quantified in the nucleus and cytoplasm prior to photobleaching, immediately after photobleaching and 5 min post-bleach (Figure 6B). The remaining fluorescence in the nucleus represents incorporated subunits that are, due to the complex size, unable to leave the nucleus. Cells that express free GFP have an immobile fraction of ~20% (Figure 6B). β1i-GFP had a higher retention rate in the nucleus with ~40% fluorescence remaining in the nuclei in transiently-transfected cells, and 50% in stably-transfected cells. As expected from the native gel analysis, β5i-GFP showed even larger immobile fractions with ~50% of the fluorescence signal remaining in the nucleus in transiently-transfected cells, and almost 90% in stably-transfected cells. Similarly, FLIP analysis of the α-subunits showed that α3-GFP was more mobile than α7-GFP, indicating less efficient incorporation into large complexes. Native gel analysis in combination with the FLIP is an easy method to examine whether tagged subunits are efficiently incorporated into proteasome complexes and thus appropriate for studying intracellular localization. Together with FRAP, it can be used to study changes in proteasome distribution patterns and kinetics.

#### Alternative Fluorescence-Labeling Strategies to Study Proteasome Kinetics

While fluorophores such as GFP allow the visualization of the total pool of tagged proteasomes, they cannot distinguish between proteasomes which are synthesized before or after a specific event or stimulus. The development of the tetracysteine (C4) motif made it possible to fluorescently label proteins at a given time-point with cell-permeable dyes (Adams et al., 2002; Martin et al., 2005), allowing fluorescent pulse-chase setups. The C4 motif, which can be genetically inserted into proteins, specifically binds the biarsenical dyes FlAsH and ReAsH. Upon binding, these dyes become green and red fluorescent, respectively. An additional advantage of these tags is their limited size. Since GFP has a molecular weight of 27 kDa while the C4-tag consists of only 12 amino acids (1.3 kDa; Griffin et al., 1998), it is likely that the C4-tag will interfere less with protein function. When C4-tagged proteins are labeled subsequently with FlAsH and ReAsH, it is possible to study two pools of the same protein that were synthesized at different time points. This method therefore can be used to determine protein turnover or protein exchange at particular intracellular sites (Gaietta et al., 2002).

**Figure 7:**
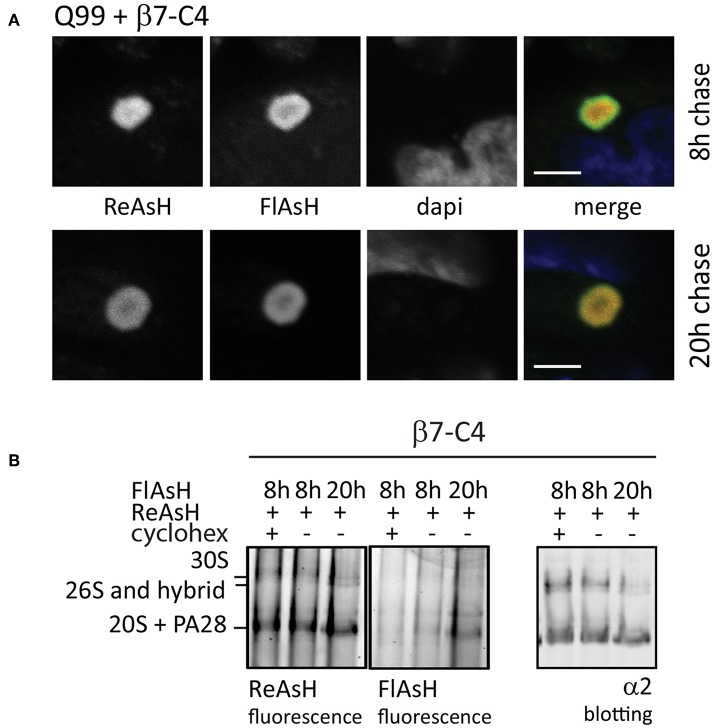
Fluorescence pulse-chase experiments to visualize proteasome dynamics. **(A)** Reversible recruitment of proteasomes into aggregates. HeLa cells were transfected with untagged Q99 peptides to initiate aggregation and co-transfected with proteasome subunit β7-C4 to visualize proteasomes. After 48 h expression, β7-C4 was stained with ReAsH followed by FlAsH labeling 8 or 20 h to specifically label the newly synthesized pool of proteasomes. Merging of the fluorescent proteasome labeling showed a partial overlap at 8 h (upper panel) and a complete overlap at 20 h afterwards (lower panel). These findings indicate that proteasomes have slow but reversible dynamics in aggregates. **(B)** Specific proteasome labeling by biarsenical dyes. Cells expressing β7-C4 were stained according to the same procedure a mentioned above. Additionally, cycloheximide was added after ReAsh staining to prevent synthesis of new C4-tagged proteasome subunits. After FlAsH staining, cells were harvested and subjected to a 3–12% native gel for complexes separation and subsequently scanned for fluorescence. Specific proteasome labeling by both ReAsH and FlAsH was confirmed since these complexes run similar to proteasome complexes that were probed with an α2-antibody. FlAsH staining intensified when labeling was performed after a longer chase period due to longer expression. When cycloheximide was added, FlAsH labeling was absent. Scale bar = 2 μm.

When studying proteasome dynamics in aggregates as observed in HD (Figure 3C), FRAP analysis suggested that these proteasomes are irreversibly sequestered into aggregates as no fluorescent recovery was observed (Holmberg et al., 2004). However, FRAP imaging usually is a relatively brief procedure used to study rapid diffusion processes, not allowing the detection of slow dynamics. To study proteasome distribution in aggregates over a longer period, we used FlAsH/ReAsH labeling in cells expressing untagged polyQ-expanded Q99 peptides to induce aggregates and co-transfected C4-tagged α7 subunits (β7-C4). Similar to mutant huntingtin fragments, polyQ peptides also form aggregates and recruit components of the UPS system (Raspe et al., 2009; Schipper-Krom et al., 2014). Cells were stained at two different time points with either ReAsH or FlasH (Figure 7A). After a 20 h chase both the old and the newly-synthesized proteasomes co-localized at the periphery of aggregates, indicating the exchange between the two proteasome pools into aggregates. This demonstrates that proteasomes are dynamically recruited into aggregates but over a longer time span, as at 8 h less co-localization is observed (Schipper-Krom et al., 2014). The specificity of the fluorescent proteasome labeling was confirmed by native gel analysis, since FlAsH- and ReAsH-labeled proteins run similar as proteasome complexes that are immunostained for α2 (Figure 7B). Additionally, FlAsH labeling was absent from 20S complexes when cycloheximide was added during the chase period, which prevents the synthesis of proteasomes and hence labeling by FlAsH. Interestingly, FlAsH incorporation into PA28-capped proteasomes but not 26S proteasomes was observed, which indicates that 26S proteasomes are far more stable complexes compared to PA28-20S proteasomes. This is in agreement with earlier observations showing a weak association between PA28 and 20S proteasomes that can be disrupted by low concentrations of salt (Ma et al., 1992). Together this illustrates the advantages of the C4-tag when studying intracellular proteasome dynamics in time dependent events.

## Discussion

Once synthesized, proteasomes are not static complexes. Altered expression of PAs, induction of cytokines and post translational modifications (PMT's) affect the function of the proteasome in the cell. Exchange of proteasome-activating caps affect protein turnover but also specific cellular processes such as cell cycle regulation, DNA transcription, and DNA repair (Ustrell et al., 2002; Chen et al., 2007; Baldin et al., 2008; Kanai et al., 2011; Levy-Barda et al., 2011; Qian et al., 2013). Interestingly, it was recently also shown that USP14, a DUB enzyme associated with the 19S complex regulates proteasome activity and substrate processing (Kim and Goldberg, 2017, 2018). Furthermore, the existence of proteasome subtypes other than the constitutive or immuno 20S particles has been shown, resulting in different cleavage specificities (Dahlmann et al., 2000; Drews et al., 2007a; Klare et al., 2007; Gohlke et al., 2014). Proteasome function is also affected by various posttranslational modifications such as glycosylation and phosphorylation, which in turn also affect activation and localization of proteasomes (Bose et al., 2004; Thompson et al., 2004; Zachara and Hart, 2004; Wu et al., 2011; Liu et al., 2014; Guo et al., 2017; VerPlank and Goldberg, 2017, 2018; Kors et al., 2019). Several kinases have already been identified to phosphorylate specific proteasome subunits (Leestemaker et al., 2017; VerPlank and Goldberg, 2017; Zhang et al., 2019). For example, phosphorylation of RPN6 increases proteasome activity and seems a very promising target to improve protein degradation in neurodegenerative diseases like amyotrophic lateral sclerosis (ALS) and Alzheimer's disease (AD) (Lokireddy et al., 2015; VerPlank et al., 2019). And finally, it was shown that proteasome interacting proteins like ZFAND and IDE also regulate proteasome activity (Stanhill et al., 2006; Lee et al., 2018; Sbardella et al., 2018). The consequences of these proteasome alterations for the distribution, dynamics, activity, and complex formation can be studied by various approaches. In this review, we have discussed a toolbox that contains tagged subunits and ABPs that label proteasome complexes. However, also other fluorescent methodologies exist, including the usage of photo-switchable fluorophores to study proteasome dynamics in living cells (Hamer et al., 2010; Zhao et al., 2016), as well as short-lived fluorescent protein substrates to quantify ubiquitin dependent proteasome activities (Table 1; Dantuma et al., 2000; Lindsten and Dantuma, 2003;Bence et al., 2005).

## Author Contributions

SS-K designed, performed research, and wrote manuscript. AS and EvB performed research. DS supervised research and revised manuscript. BF generated material and supervised manuscript. HO generated materials. ER designed and supervised research and revised manuscript.

### Conflict of Interest Statement

The authors declare that the research was conducted in the absence of any commercial or financial relationships that could be construed as a potential conflict of interest.
